# Regional Heritability Mapping of Quantitative Trait Loci Controlling Traits Related to Growth and Productivity in Popcorn (*Zea mays* L.)

**DOI:** 10.3390/plants10091845

**Published:** 2021-09-06

**Authors:** Gabrielle Sousa Mafra, Janeo Eustáquio de Almeida Filho, Antônio Teixeira do Amaral Junior, Carlos Maldonado, Samuel Henrique Kamphorst, Valter Jário de Lima, Divino Rosa dos Santos Junior, Jhean Torres Leite, Pedro Henrique Araujo Diniz Santos, Talles de Oliveira Santos, Rosimeire Barboza Bispo, Uéliton Alves de Oliveira, Vitor Batista Pinto, Alexandre Pio Viana, Caio Cezar Guedes Correa, Sunny Ahmar, Freddy Mora-Poblete

**Affiliations:** 1 Centro de Ciências Agrárias, Universidade Estadual da Região Tocantina do Maranhão, R. Godofredo Viana, 1300, Imperatriz 65900-000, Brazil; gabrielle.smafra@yahoo.com.br; 2 Bayer, Estrada da Invernadinha, 2000, Coxilha 99145-000, Brazil; janeo.filho@bayer.com; 3 Laboratory of Plant Breeding, Center of Agricultural Science and Technology, Darcy Ribeiro State University of Northern Rio de Janeiro, Av. Alberto Lamego, 2000, Campos dos Goytacazes 28013-602, Brazil; samuelkampho@hotmail.com (S.H.K.); valter_jario@hotmail.com (V.J.d.L.); juniorifagro@gmail.com (D.R.d.S.J.); torresjhean@gmail.com (J.T.L.); phsantos2004@yahoo.com.br (P.H.A.D.S.); tallesdeoliveira@live.com (T.d.O.S.); rosimeirebarboza1@hotmail.com (R.B.B.); uelitonalves2011@hotmail.com (U.A.d.O.); vvitorbp@gmail.com (V.B.P.); pirapora@uenf.br (A.P.V.); caiocagronomo@gmail.com (C.C.G.C.); 4 Instituto de Ciencias Agroalimentarias, Animales y Ambientales, Universidad de O’Higgins, San Fernando 3070000, Chile; cmaldo1782@gmail.com; 5 Institute of Biological Sciences, University of Talca, 1 Poniente 1141, Talca 3460000, Chile; sunnyahmar13@gmail.com (S.A.); morapoblete@gmail.com (F.M.-P.)

**Keywords:** candidate genes, linkage disequilibrium, genomic regions, regional heritability mapping, single nucleotide polymorphism

## Abstract

The method of regional heritability mapping (RHM) has become an important tool in the identification of quantitative trait loci (QTLs) controlling traits of interest in plants. Here, RHM was first applied in a breeding population of popcorn, to identify the QTLs and candidate genes involved in grain yield, plant height, kernel popping expansion, and first ear height, as well as determining the heritability of each significant genomic region. The study population consisted of 98 S1 families derived from the 9th recurrent selection cycle (C-9) of the open-pollinated variety UENF-14, which were genetically evaluated in two environments (ENV1 and ENV2). Seventeen and five genomic regions were mapped by the RHM method in ENV1 and ENV2, respectively. Subsequent genome-wide analysis based on the reference genome B73 revealed associations with forty-six candidate genes within these genomic regions, some of them are considered to be biologically important due to the proteins that they encode. The results obtained by the RHM method have the potential to contribute to knowledge on the genetic architecture of the growth and yield traits of popcorn, which might be used for marker-assisted selection in breeding programs.

## 1. Introduction

Maize (*Zea mays* L.) is among the most important cereal crops in the world in addition to wheat and rice [1], which has been widely cultivated due to its nutritional composition, versatility, and broad adaptability. Both the land area used for maize grain production and the amount of maize produced per unit area in Brazil has increased in recent years by 25% and 60%, respectively [2], which makes Brazil the third largest maize grain producer worldwide. Popcorn is a special type of maize primarily used for human consumption due to its exceptional nutritional and functional properties, i.e., the average dietary fiber content of 17.79%, and low-calorie count when prepared without oil or fat [3]. The economically most important traits evaluated in popcorn breeding programs are grain yield (GY) and popping expansion (PE) [4]. However, the selection of cultivars based on traits such as plant height (PH) and ear height (EH) have important effects on plant lodging in intensive maize cultivation systems [1]. The yield (GY and PE) and growth (PH and EH) traits of maize are usually quantitatively inherited, and their genetic basis is controlled by the interaction between multiple genetic and environmental factors [1,5,6,7].

A successful tool to explain the genetic basis of complex traits in association studies, which allow the identification of quantitative trait loci (QTLs) based on the significant associations between genotypic markers and phenotypic data [8]. The identification of QTLs related to PE and GY has been reported in several studies, for example, simple sequence repeat (SSR) and single-nucleotide polymorphism (SNP) markers [5,8,9,10,11,12]. Thakur et al. [10] identified three QTLs associated (using SSR markers) with the popping volume, which covers 78% of total phenotypic variance. Dell’Acqua et al. [12] identified three suggestive QTLs for GY, of which the locus on the short arm of chromosome 6 was defined as a major QTL, accounting for 13% of the variance in GY. The identification of QTLs related to PE has mainly been reported using SSR markers, presenting a reduced number of identified QTLs. This fact may be related to the low density of markers used. The use of high-throughput genotyping technologies has filled this gap, as they are more accurate and allow the deeper dissection of genomes of species such as popcorn [13].

The SNP has been widely used in association studies for the detection of a large number of QTLs and candidate genes involved in the yield and growth [8,14,15]. Despite their wide usage, association studies have been criticized, since according to certain studies, they are inefficient in detecting the total genetic variation of complex traits [16,17]. In this sense, some studies have reported that SNPs markers associated with a complex trait can typically capture only a small proportion of genetic variance [10], which has been called the “missing heritability” problem [18].

Research in human genetics has shown that multiple independent loci with different frequencies and allelic effects are commonly located in the same gene region or short-segment regions [19,20]. These loci may be undetectable by single SNP analyses since this type of analysis is not sufficiently sensitive to identify relatively small individual allelic effects, even if the cumulative effect of the entire locus on the trait variance is high [17].

To overcome this drawback and capture most of the genetic variance that cannot be captured by association studies, recent studies have proposed a method called regional heritability mapping (RHM) [16,17], which facilitates the detection of the genetic variation related to each genome segment [16,17,18,21]. The RHM method uses a relationship matrix between individuals based on common and rare SNP information from small regions of genomes to estimate the trait variance explained by each region and localize variation [22]. In RHM, the genomic and regional heritability is estimated using a mixed model based on the restricted maximum likelihood (REML) and two components of variance; one is attributed to the entire genome and the other to a specific genomic region [17]. It is suggested here that an approach with the RHM methodology could identify common and rare variants involved in the expression of complex traits, e.g., of grain yield and popping expansion in popcorn populations.

To date, studies based on RHM analysis are not well consolidated for plant species, since most research using this type of methodology has been developed to identify QTLs related to complex traits in humans and animals [17,22,23]. Thus far, few studies with plant species are available in the literature. Some examples are the comparisons of the methodologies of association studies and RHM in *Eucalyptus* [18], in common bean (*Phaseolus vulgaris* L.) [24], and cassava (*Manihot esculenta* Crantz) [25]. Resende et al. [18] compared an association analysis and RHM in complex traits of *Eucalyptus* and observed that RHM allowed the identification of 13 more QTLs than association analysis. In addition, the authors observed that RHM outperformed the genome-wide association study analysis for all traits evaluated, capturing, in general, two or three times the amount of genomic heritability. Similarly, RHM allowed a greater proportion of genomic heritability to be explained compared to association analysis, in accessions of *Phaseolus vulgaris* L. [24].

To the best of our knowledge, this is the first study to apply RHM to understand the genetic basis of complex traits in popcorn. The objectives of this study were to identify genomic regions and candidate genes associated with growth (plant height and ear height), popping expansion, and grain yield of popcorn, as well as to examine the genomic heritability attributed to these traits, using regional heritability mapping with a mixed model approach.

## 2. Results

**Figure 1:**
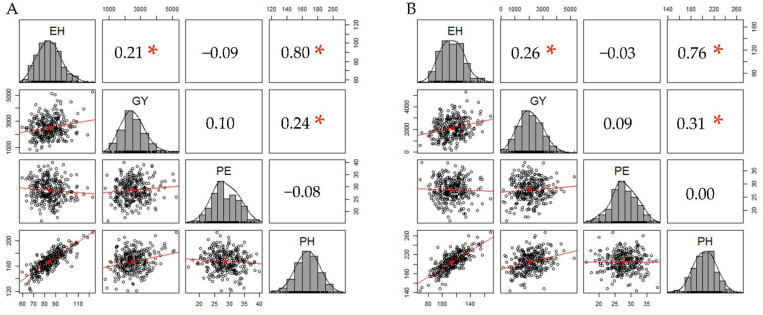
Plot representing phenotypic distribution and correlation for EH (ear height), GY (grain yield), PE (kernel popping expansion), and PH (plant height) traits, evaluated in (**A**) Campos dos Goytacazes (ENV1) and (**B**) Itaocara (ENV2). The diagonal line of the plot shows the histograms and the distribution of the observed phenotypes values. The lower off-diagonal is the scatterplot between the four traits, whereas the upper off-diagonal represents the correlation value between traits. Significant correlations (*p*-value < 0.001) are tagged with an asterisk.

The average values for ear height (EH), plant height (PH), and grain yield (GY) differed between the two environments, with EH and PH higher values in ENV2 (36% and 18%, respectively) to ENV1, while GY in ENV1 was 20% higher than ENV2 (Supplementary Table S1). On the other hand, PE did not show changes between ENV1 and ENV2 (28.7 and 27.8, respectively). A histogram for the four traits and correlation coefficients between pairs of traits is shown in Figure 1. The normality hypothesis was significant in all traits in both environments. The coefficients of correlation among EH, GY, and PH were positive and statistically different from zero (*p*-value < 0.001) in ENV1 and ENV2, with values of *r* = 0.21 and 0.26 for EH-GY (ENV1 and ENV2, respectively), *r* = 0.80 and 0.76 in EH-PH (ENV1 and ENV2, respectively) and *r* = 0.24 and 0.31 in PH-GY (ENV1 and ENV2, respectively).

### 2.1. SNP Data, LD, and Detection of Associations via RHM

21,442 polymorphic loci were identified among the maize plants from the breeding population throughout the Capture Seq method [26]. Individuals with a data loss of upper to 10% were removed resulting in 196 individuals. Subsequently, the SNPs were filtered to 10,507 by selecting the loci with MAF (Minor Allele Frequency) upper 0.05 and with less than 5% of missing data. The molecular markers were aligned with the 10 chromosomes of the reference genome of maize and presented a wide distribution throughout the genome. The average number of SNPs per chromosome was 1051, ranging from 778 on chromosome 6 to 1598 on chromosome 1. 

**Figure 2:**
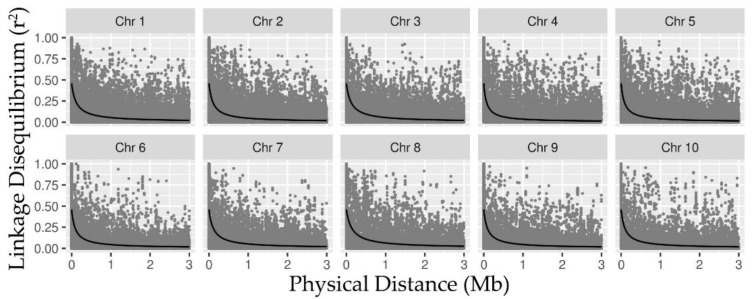
Decay of linkage disequilibrium (LD) of all SNPs (10.507) in the ten chromosomes estimated by *r*^2^ (y-axis) along with physical distance in Mb (x-axis).

The pattern of LD was estimated for each chromosome considering all SNPs belonging to the same chromosome. The slowest LD decay was observed in chromosome 8, in which half of the decay occurred at ~151 Kb (kilobase pairs), while chromosome 4 decreased the fastest, in which half of the decay was observed at ~76 Kb. The average half of the LD across the chromosomes was ~110 Kb ± 6.82 (Figure 2). This result is consistent with that previously reported by [27], which observed that with the same population, half of the LD occurred in ~107 Kb.

**Figure 3:**
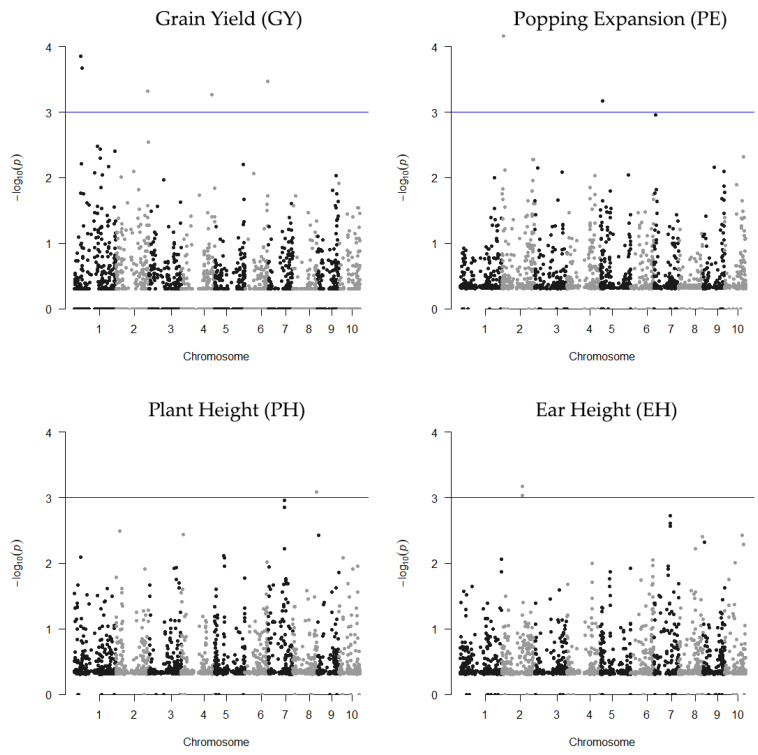
Manhattan plots of the statistical significance of the genomic regions associates with the GY, PE, PH, and EH traits in Campos dos Goytacazes (ENV1). The x-axis shows chromosomal positions and the y-axis shows −log_10_ *p*-values. The blue horizontal line represents the genome-wide significance threshold (*p*-value = 1 × 10^−3^).

**Figure 4:**
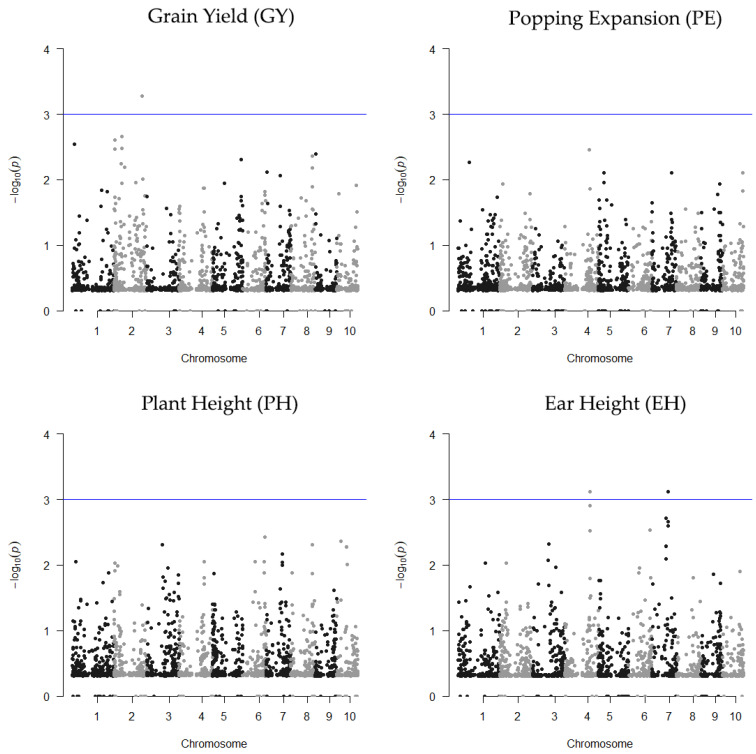
Manhattan plots of the statistical significance of the genomic regions associates with the GY, PE, PH, and EH traits in Itaocara (ENV2). The x-axis shows chromosomal positions and the y-axis shows −log_10_ *p*-values. The blue horizontal line represents the genome-wide significance threshold (*p*-value = 1 × 10^−3^).

Seventeen QTLs were found by RHM in ENV1, each encompassing between 2 and 8 SNPs on chromosomes 1, 2, 4, 5, 6, and 8, while in ENV2, five QTLs were mapped, which ranged between 2 and 9 SNPs on chromosomes 2, 4 and 7 (Figure 3 and Figure 4). In ENV1, the regional QTLs for EH and PH were located on individual chromosomes, while QTLs for GY and PE were mapped on several different chromosomes (Figure 3 and Supplementary Table S2). Notably, three regionals QTL (two associated with GY and one associated with PE) were detected at the same positions as the QTLs detected by Mafra et al. [8] using SNP-based mixed-model association (Supplementary Table S2). In ENV2 (Figure 4 and Supplementary Table S2), for trait GY, regional QTLs were identified on individual chromosomes, while for EH, they were mapped on different chromosomes. Heritability varied between 0.079 (EH) and 0.93 (GY) in ENV1 and between 0.078 (GY) and 0.14 (EH) in ENV2. The genome-wide distribution of regional heritability QTLs mapped by RHM along the 10 maize chromosomes are shown in Figure 3 and Figure 4, displaying significant QTLs (*p*-value ≤ 0.001, or −log_10_ (*p*-value) ≥ 3) found in ENV 1 and ENV 2, respectively.

### 2.2. Gene Identification in QTL Regions

As the main findings by RHM in both environments, possible candidate genes were identified based on the physical position of reference genome B73 (Table 1 and Table 2) and regions significantly associated with the traits. Forty-six candidate genes were found within these regions, of which thirty-seven candidate genes were found to be associated with GY, PE, and PH of ENV1, and nine to the EH and GY traits in ENV2. Notably, in the PE (ENV1) and PH (ENV1), the regions 13388849-13488849 (chromosome 2) and 171723438-171823438 (chromosome 8) presented the largest number of associated candidate genes (8 and 7, respectively).

## 3. Discussion

### 3.1. Linkage Disequilibrium and Heritability Captured by RHM

Half of the LD decay varied among chromosomes (76–151 Kb), which suggests differences in their rate of recombination [18]. The linkage disequilibrium in allogamous species, e.g., in maize, decays over relatively shorter distances than in autogamous species [62]. However, in an evaluation of 64 corn lines [63], it was observed that the average LD decay length in all of them was 80–100 kb [63]. Similarly, was observed that in 144 maize lines, the average LD decay for all chromosomes was around 200 kb [64].

Heritability estimated based on RHM was low for most traits and in both environments (Supplementary Table S2), with ENV2 presenting the lowest heritability at 0.078 for GY.

In ENV1, for GY—one of the key traits of interest in popcorn—a series of heritability values were obtained, which ranged from 11% to 93%. This variation may be due to population effects, environmental factors, or experimental precision [24], or, more likely, genome partitioning caused by the RHM methodology. Moreover, simulating data of parental lines, genotypes, and phenotypes F_1_ and F_2_ and using the maximum likelihood approach by interval mapping for low heritability QTLs and high SNP density, obtained a value of approximately 95% heritability for GY when using a sample number of 200 [65].

Previous studies have reported that the trait heritability of GY varies from low to moderate [66,67], indicating a lower additive genetic control and high environmental influence. However, other research has claimed that the heritability of GY is high and significant [68,69] and suggested, therefore, that these values depend on the population under study. In the UENF-14 population intrapopulation recurrent selection program, it was observed that heritability remained stable in cycles 4, 5 and 6 (45.97%, 51.94%, and 45.04%, respectively) [70]. In addition, a heritability of 91% for GY was observed in cycle 9 of the same population [71]. In research carried out by Resende and collaborators [24], the estimated genomic heritability captured a relatively large proportion (72%) of the total heritability of the traits. This was useful in identifying favorable alleles for GY of *Phaseolus vulgaris* L, demonstrating that the RHM method was effective in detecting the hereditary portion of the trait in the population.

On the other hand, in ENV1, the inheritable proportion of total variability of PH and EH traits was the lowest (Supplementary Table S2). Some hypotheses can be raised in this regard, e.g., the fact that the traits EH and PH are directly correlated and have a high heritability [7]. However, when partitioning the genome, as in the RHM method, the values within each genomic window may be too low to be detected by the analysis. The same is not true for GY, for which the highest heritability values were recorded by RHM, and which, despite the strong environmental influence on it [66,67], has been shown to remain stable in successive cycles of intrapopulation recurrent selection in the population sampled here [70,71].

### 3.2. RHM Analysis and Candidate Genes

Among the QTLs observed in ENV1, some genes were considered to be biologically important due to their encoded proteins and proven participation in the studied traits.

The GRMZM2G069618 gene reported to be involved in the expression of GY encodes the protein containing the tetratricopeptide repeat domain (TPR). The TPR is involved with numerous outstanding functions in plant organisms, for example, hybrid sterility in rice [72], hormonal signaling and stress [73], root development [29], and failure of endosperm development, with a consequent reduction in seed number [74]. In addition, in rice, TPR may be involved in amylose content, grain appearance, physical and chemical properties [75], and grain size and starch quality [76]. The results found in this study corroborate the hypotheses of TPR can be directly involved with the expression of kernel weight in popcorn, consequently influencing the GY trait.

The GRMZM5G846057 gene, linked to the GY trait, is related to the AP2 domain (AP2) protein. APETALA2/ethylene proteins (AP2/EREBPs) are the main regulators of responses to plant development, growth, and stress [77,78]. AP2/EREBP genes play a crucial role in responding to various environmental stresses in cotton [77]. ERF transcription factors are AP2/EREBP proteins that contain only one AP2 domain and constitute the largest subfamily of the AP2/EREBP family [78]. Although a direct relationship has not been identified between GRMZM5G846057 and GY, this trait seems to be directly influenced by abiotic stress, as reported in several studies [79,80,81,82,83]. Therefore, it is possible to suggest that the trait GY is related to responses to stresses and when there is a reduction in grain yield, there is also overexpression of the GRMZM5G846057 gene, which codes AP2/EREBP, related to stresses in plants.

The GRMZM2G414114 gene, which is related to the expression of the protein DNAj (??) domain//TCP family transcription factor//Transposase-associated domain, is involved with grain yield. This protein, called TCT, refers to transcription factors observed in plants and is involved in the growth and development of several species [84], including the growth of axillary organs and corn ear formation [34], formation of the shoot meristem and shoot development of Arabidopsis [85], and development or ripening of tomato fruits [86]. Thus, the involvement of TCP in the development process of popcorn kernels can be suggested, since kernel weight is directly related to the total trait yield, represented here by the acronym GY.

The GRMZM2G051958 gene, responsible for coding the enzyme phosphoenolpyruvate carboxykinase ATP (PEPCK), is involved in the expression of the trait PE. Studies report that this protein plays a significant role in the photosynthesis of C4 plants of the Poaceae family, in which corn is inserted [87], as well as the increase in the photosynthetic rate of wheat plants with a significant increase in the grain yield of the species [88,89]. In addition, research reports the importance of the enzyme phosphoenolpyruvate carboxykinase in the development of seeds of species such as peas (Pisum sativum), highly involved in the coating of seeds and cotyledons [38]. An overexpression of PEPCK in common bean seeds was found to raise protein accumulation in the organ [90].

GRMZM2G069618, GRMZM2G414114, and GRMZM2G051958 encode proteins that would directly affect GY by regulating the weight and size of grains, increase in the photosynthetic rate, and the physical and chemical properties of popcorn. Moreover, these genes are involved in the growth and development of plants, and the development process of popcorn kernels, key aspects in the GY. Particularly, GRMZM2G051958 has shown a significant increase in the grain yield in wheat through an increase in the photosynthetic rate. On the other hand, GRMZM5G846057 encodes a protein AP2, which regulates the GY in conditions of abiotic stress. In this sense, Xu et al. [91] overexpressed the ZmCBF3 (a maize AP2/ERF-type transcription factor) in rice plants, and they showed that tolerance to drought stress was improved without not affect the grain yield. Therefore, advancing the knowledge of this gene in popcorn can help preserve GY under conditions of drought stress, an important aspect considering current climatic conditions.

A relationship between PH and the GRMZM2G133175 gene, which encodes a cysteine-rich TM module stress tolerance (CYSTM), was observed. The cysteine-rich transmembrane module CYSTM, commonly found in eukaryotes, is composed of a small family of molecular proteins [92]. The CYSTM genes exhibit a broad and constant expression pattern that may play vital roles in plant growth and development [92]. Moreover, this module was responsible for conferring tolerance to heavy metals such as cadmium and copper in *Digitaria ciliaris* and *Oryza sativa* [50], and resistance to abiotic stress in Arabidopsis thaliana [92]. Xu et al. [92] suggest that the CYSTM family, as newly discovered small peptides, plays multiple roles in plant growth and development, especially in response to abiotic stresses.

In ENV2, the GRMZM2G060630 gene, which encodes the mitochondrial phosphate transporter (MPT) member 3 protein, was related to EH. Mitochondrial phosphate transporters have been identified as responsible for plant responses to salt stress *in Arabidopsis thaliana* [93] and indispensable for the growth and development of plants of this species [58]. This led to the assumption of their direct involvement in growth related to the insertion height of the first ear (EH) in popcorn plants. Jia et al. [58] suggested that MPT3 played important role in regulating plant growth and development in Arabidopsis.

The RHM approach to the analysis of SNP data has the potential to explain part of the missing heritability by capturing QTL variance from small regions of the genome, which escape the standard association, single SNP-based studies. For example, Mafra et al. [8], in this same population, observed that heritability estimates for the SNP markers allowed them to capture <0.01 in GY for ENV1, while the RHM method registered a heritability between 0.11 and 0.93, capturing the missing heritability in this trait. Therefore, the RHM strategy used in the present study was proven to be useful and robust, complementary to other association analyses. Moreover, RHM analysis identified QTLs and candidate genes related to traits of interest, which can be incorporated into breeding programs and genomic selection strategies in maize.

## 4. Materials and Methods

### 4.1. Study Population and Phenotypic Evaluations

The study was carried out in August 2016, at the Research Station of the Antônio Sarlo State College of Agriculture (21°43′14.8” S, 41°20′38.3” W), in the county of Campos dos Goytacazes (Rio de Janeiro—RJ) (hereafter ENV1), and on an experimental field of the State University of Northern Rio de Janeiro—UENF (21°38′45.2 S 42°03′16.3” W), on the Ilha Barra do Pomba, county of Itaocara (Rio de Janeiro—RJ) (henceforth ENV2). The ENV1 has a dry tropical climate with an average annual temperature around 24 °C and annual precipitation of 1112 mm [94], while ENV2 has a warm climate with an average annual temperature around 22.5 °C and annual precipitation of 1041 mm [95]. 

The study population consisted of 98 S1 families derived from the 9th recurrent selection cycle (C-9) of the open-pollinated variety UENF-14 [96,97,98], which was developed from five cycles of recurrent selection of the population UNB-2U. The UNB-2U was derived from UNB-2 after two mass selection cycles in Campos dos Goytacazes, Rio de Janeiro, Brazil [99]. After five cycles of intrapopulation recurrent selection of UNB-2U, the cultivar UENF-14 was launched [96], and today it is in the 9th cycle of recurrent selection (C-9), used in this research.

The cycles of recurrent selection to obtain the studied population were obtained through the strategies described in Table 3, with their respective gains in PE% and GY%.

The tests were designed in incomplete blocks with three orthogonal repetitions. Seeds of each family were sown in a 5 m long row, at a spacing of 0.90 m between rows and 0.20 m between plants. When necessary, cultural treatments were carried out as recommended for the crop.

At both locations, the main traits of agronomic interest for the crop were measured: grain yield (GY, Kg/ha), kernel popping expansion (PE, mL/g), plant height (PH, cm), and the first ear height (EH, cm). GY was measured after the threshing process of all ears in the plot, where the weight (weighed on a precision scale) in Kg of the kernels per plot was transformed to kg ha^−1^. PE was measured by weighing 30 g kernels per plot on a precision scale; then, these were inserted into kraft paper bags and microwaved for 2 min 20 s. Afterward, the popped kernels were filled in 2000 mL beakers, and the resulting volume was divided by the initial kernel weight (30 g of grains). PH was measured from the soil level to the flag leaf insertion in six healthy plants per plot (with a ruler). Similarly, EH was measured in six healthy plants per plot, from the soil level to the insertion of the first ear (with a ruler). Data from the phenotypic evaluations are shown in Supplementary Table S1. The phenotypic data were adjusted using the lme4 package [103] in R software [104], by the following mixed model:

(1) $$y=X\beta_{1}+Z_{1}b+Z_{2}p+e$$

where y is the phenotype vector of a given trait; $\beta_{1}$ is the vector with fixed effects, including intercept, repetition, and covariates such as the number of plants per plot, counted immediately after thinning and kernel moisture, for the traits GY and PE [8]; b is a vector of block effects within replications; p is the progeny effect, assumed as fixed to estimate the adjusted means (with package LSMeans) [105]; e is the vector of residual effects of the model. X is the incidence matrix of systematic fixed effects, and *Z*_1__,2_ are incidence matrices of random effects.

### 4.2. Genotyping

Genomic DNA was extracted from the young leaves of 200 plants, from the breeding population, using the standard CTAB method of Doyle and Doyle [106] with modifications. The collected DNA samples were sent to the company Rapid Genomics LLC for sequencing by the Capture Seq method [26], with 5000 well-distributed probes in the maize reference genome. These probes are selected and obtained from known reference regions, available in the database. In the Capture Seq method, the genomic DNA is fragmented to hybridize with barcode adapters. After the probes have captured the target fragments amplified, the libraries are assembled for sequencing. Subsequently, the sequencing data file is filtered by individual barcodes and is aligned by the reference genome.

### 4.3. Genetic Aspects of the Population

From the panel of 10,507 SNPs and 196 individuals, the Genetic Relationship Matrix (GRM) was calculated, with the rrBLUP package [107], based on the algorithm of VanRaden [108]. This same panel of genotypic data was used to estimate the linkage disequilibrium (LD), based on *r*^2^ statistics, using PLINK software [109]. To analyze the LD decay across the genome, the nonlinear model proposed by Hill and Weir [110] was fitted, using the nlm function of the R language [104].

### 4.4. Regional Heritability Mapping

Regional heritability mapping provides heritability estimates for genomic segments containing common and rare allelic effects [24]. The RHM model presented in the following equation was adjusted using the Regress package [111] in R software [104].

(2) $$y=X\beta_{1}+Z_{1}b+Z_{2\;}r+Z_{3\;}u+e$$

where 𝑦 is the vector with the phenotypes of a given trait; 𝛽_1_ is the vector with fixed effects—intercept, repetition and covariables such as the number of plants per plot, counted immediately after thinning and kernel moisture for the traits GY and PE; *b* is the vector of block effects; *r* is the vector of random regional genomic additive effects; *u* is the vector of polygenic effects; *e* is the residual effect vector of the model. *X* is the incidence matrix of systematic fixed effects, and *Z*_1,2,3_ are incidence matrices of random effects.

The following was assumed:

(3) $$b|\sigma_{b}^{2}\sim N\left(0,I\sigma_{b}^{2}\right);\;r|\sigma_{r}^{2}\sim N\left(0,G_{reg}\sigma_{r}^{2}\right);\;u|\sigma_{u}^{2}\sim N\left(0,G\sigma_{u}^{2}\right);\;e|\sigma_{e}^{2}\sim N\left(0,I\sigma_{e}^{2}\right);$$

(4) $$\mathrm{cov}\left(u,b^{\prime }\right)=\mathrm{cov}\left(u,e^{\prime }\right)=\mathrm{cov}\left(b,e^{\prime }\right)=0$$

where *G* is the genomic matrix of kinship, calculated with the rrBLUP package [107], based on the algorithm of VanRaden [108]; *Greg* is a matrix similar to *G*, but uses a subset of matrix *W* (*W* is the SNP marker incidence matrix assuming *W* ⊂ (−1, 0, 1)). These subsets were determined by 100 kb long genomic “windows” or “regions”, with overlapping stretches of 50 kb (for example, the first three regions are 0–100, 50–150, and 100–150 kb), corresponding to the estimated LD—see Results section); Ib and In are identity matrices with the same order as that of the number of incomplete blocks and number of observations, respectively; $\sigma_{b}^{2}$, $\sigma_{r}^{2}$, $\sigma_{u}^{2}$, and $\sigma_{e}^{2}$ are the components of variance associated with *b*, *r*, *u*, and *e*, respectively. These components were estimated by REML, using the average information (AI) algorithm. RHM was adjusted by the Regress package [111], in language R 3.2.3 [103], according to the procedure proposed by Resende [18]. Significance testing was based on a likelihood ratio test statistic (LRT) with *p*-value ≤ 0.001, or −log_10_ (*p*-value) ≥3.

For each segment of the genomic window, the regional heritability was determined by the following equation:

(5) $$\mathrm{cov}\left(u,b^{\prime }\right)=\mathrm{cov}\left(u,e^{\prime }\right)=\mathrm{cov}\left(b,e^{\prime }\right)=0$$

where $\sigma_{r}^{2}$, $\sigma_{g}^{2}$, and $\sigma_{e}^{2}$ are the components of variance associated with *r*, *g*, and *e*, respectively.

### 4.5. Identification of Candidate Genes

The QTLs identified by RHM analysis were subjected to gene identification analysis, using the public maize genome data set, based on the reference genome B73 version 3 (gene annotation in the B73 Zea mays AGPv3 assembly) [112]. Functional gene annotations were identified using the Phytozome genome browser (https://phytozome.jgi.doe.gov/pz/portal.html, (accessed on 10 November 2020)). The gene search regions were defined according to the start and end position of each region. The genes detected within these regions were defined as candidate genes.

## 5. Conclusions

RHM allowed us to combine common and rare variants within the same analysis, which provided valuable information on the genetic architecture of yield and growth traits. It was confirmed that RHM has the potential to explain some portion of missing heritability by capturing variance caused by QTL with low MAF and multiple independent QTL within a region, as determined for grain yield. Moreover, the genomic regions identified by RHM allowed further examination towards candidate genes discovery. In this regard, a total of 22 genomic regions were identified in both tropical environments, which may be related to the adaptability of the genotypes to each environment. Subsequent gene identification study revealed associations with several genes with a known function in plants. Forty-six candidate genes were associated with these genomic regions, of which seven are considered to be biologically important, encoding proteins that participate in plant development processes, and suggesting different roles in the performance of functions related to popcorn growth and yield. The QTLs and candidate genes detected in this study broaden the knowledge on the molecular basis of these important agronomic traits and could potentially help to increase the efficiency of popcorn breeding programs, creating new opportunities for the development and selection of elite breeding genotypes.

## Figures and Tables

**Table 1:** Candidate genes detected by regional heritability analysis for four traits of interest for popcorn in environment ENV1.

Trait	* Chrom.	Minor-Major Position (Base Pairs)	Gene ID	Annotation	Putative Function
GY	1	48905387-49005387	GRMZM2G003984	Lon-like ATP-dependent protease	Seed germination [28]
GY	1	56805387-56905387	GRMZM2G069618	TPR domain-containing protein	Plant development [29]
GY	1	56855387-56955387	GRMZM2G353147	GTP diphosphokinase/stringent factor	Plant fertilization [30]
GY	6	165623194-165723194	GRMZM5G846343	Protein of unknown function	** NA
			GRMZM5G846057	AP2 domain (AP2)	Growth and development of plant tissues [31]
GY	2	225088849-225188849	GRMZM2G037993	Respiratory burst oxidase homolog protein a-related	Root development [32]
			GRMZM2G015945	Respiratory burst oxidase homolog protein b	Pathogen resistance [33]
GY	2	225138849-225238849	GRMZM2G414114	Dnaj domain (dnaj) //TCP family transcription factor (TCP)//transposase-associated domain	Growth of axillary organs and corn ear formation [34]
			GRMZM2G114948	Plant protein of unknown function	** NA
			GRMZM2G023328	Arginine and glutamate-rich protein 1 (ARGLU1)	** NA
			GRMZM2G023585	Hira-interacting protein 3	** NA
GY	4	219528603-219628603	GRMZM2G043242	Zinc finger cw-type coiled-coil domain protein 3	Plant immunity to disease [35]
GY	4	219578603-219678603	GRMZM2G109159	Reticulon-like protein	Seed filling [36]
PE	2	13388849-13488849	GRMZM5G886913	Predicted membrane protein	Essential for many functions [37]
			GRMZM2G051958	Phosphoenolpyruvate carboxykinase ATP	Involved in the coating of seeds and cotyledons [38]
			GRMZM2G354053	Myosin heavy chain-related//	Movement of the Golgi complex and mitochondria in plant cells [39].
			GRMZM5G866405	Isoleucine--tRNA ligase/Isoleucyl-tRNA synthetase	** NA
			GRMZM5G899760	GDP dissociation inhibitor (GDI)//transcription initiation factor IIA,	Pollen germination and tube growth [40]
			GRMZM2G059791	2-keto-3-deoxy-l-rhamnonate aldolase	** NA
			GRMZM2G359331	Myosin heavy chain-related	Movement of the Golgi complex and mitochondria in plant cells [39]
			AC195235.3_FG003	Phosphoglyceromutase	Concentration increase in iron-deficient *Cucumis sativus* L. roots [41]
PE	5	13560296-13660296	GRMZM2G461948	Ubiquitin-protein ligase	** NA
			AC194618.2_FG008	DNA(??) homolog subfamily c member	Cold tolerance in transgenic tomato plants [42]
PE	5	13560296-13660296	GRMZM2G461959	Serine/threonine-protein phosphatase pp2a-1 catalytic	Expressed in stems, flowers, and roots of *Oryza sativa* [43]
			GRMZM2G461936	Translation initiation factor 2C (eif-2C)	Regulation of various aspects of plant development and their interactions with the environment [44]
			GRMZM2G161242	Protein Y55F3AM.3, isoform a	** NA
			GRMZM2G161222	Serine/threonine-protein phosphatase 2a 57 kDa regulatory subunit β’ alpha isoform	Control of biotic and abiotic stress responses in plants [45]
			GRMZM2G148130	Ubiquitin-conjugating enzyme E2 16	Maintenance of normal maize growth under stress conditions [46]
PE	5	13610296-13710296	GRMZM2G148098	Homeobox protein transcription factors	Maintenance of adequate meristem and organ initiation [47]
			GRMZM2G122185	Pre-mRNA splicing factor	Temperature signaling in plants [48]
PH	8	171723438-171823438	GRMZM2G133249	Insulysin (IDE, ide)	Beta-amyloid (Aβ) degradation [49]
			GRMZM2G562929	Proteasome subunit alpha type-4	** NA
			GRMZM2G133175	Cysteine-rich TM module stress tolerance (CYSTM)	Tolerance to heavy metals, such as cadmium and copper [50]
			GRMZM2G133029	Aspartyl protease family protein	Plant defense against fungi [51]
			GRMZM2G132991	Conserved oligomeric Golgi complex subunit 1	Resistance penetration in barley by the barley mildew fungus [52]
			GRMZM2G132978	Pthr36737:sf1-expressed protein	** NA
			GRMZM2G434363	Protein kinase domain (Pkinase)//salt stress response/antifungal (stress–antifungal)	Response to salt stress [53]

* Chrom: chromosome; ** NA: not applicable.

**Table 2:** Candidate genes detected by regional heritability analysis for four traits of interest for popcorn in environment ENV2.

Trait	* Chrom.	Minor-Major Position (Base Pairs)	Gene ID	Annotation	Putative Function
EH	7	115031917-115131917	GRMZM2G022095	rRNA N-glycosylase/rRNA N-glycosidase	Antiviral, antifungal, and insecticidal activities and their expression in plants are increased under stress conditions [54]
EH	7	115081917-115181917	GRMZM5G837058	Golgi SNAP receptor complex member 1-1	Transport from Golgi complex to the endoplasmic reticulum [55]
			GRMZM2G071059	CCR4-NOT transcription complex subunit 7/8 (CNOT7_8, CAF1, POP2)	Responses to abiotic stress [56]
EH	4	178478603-178578603	GRMZM2G170313	Prolyl-tRNA synthetase associated domain-containing protein 1-related	** NA
			GRMZM2G473016	Ring zinc finger protein	Increased stomatal opening [57]
			GRMZM2G060630	Solute carrier family 25 (mitochondrial phosphate transporter), member 3	Normal growth and development of *Arabidopsis* [58]
			GRMZM2G060554	Remorin, C-terminal region (Remorin_C)	Resistance to helminthosporium in corn [59]
			GRMZM2G356046	Mannan endo-1,4-beta-mannosidase (MAN)	Seed germination [60]
GY	2	200888849-200988849	GRMZM2G024622	RNA polymerase II ctd phosphatase	Increased tolerance to thermal stress [61]

* Chrom: chromosome; ** NA: not applicable.

**Table 3:** Genetic gains across recurrent selection cycles in the UNB-2U population.

Cycle	Reference	Strategy	PE (%) *	GY (%) *
1°	Daros et al. [97]	Full-sib families	10.39	4.69
2°	Daros et al. [98]	Inbred families (S_1_)	17.8	26.95
3°	Santos et al. [99]	Half-sibling families	7.16	10.00
4°	Freitas Júnior et al. [100]	Full-sib families	10.58	7.71
5°	Rangel et al. [101]	Full-sib families	6.01	8.53
6°	Ribeiro et al. [69]	Full-sib families	10.97	15.30
7°	Freitas et al. [102]	Full-sib families	5.11	7.78
8°	Guimarães et al. [71]	Full-sib families	3.61	4.60

Cycle: selection cycle; Reference: started by the first author of the publication of the article referring to the respective cycle; PE: kernel popping expansion; GY: grain yield; * Genetic gains (in percentage) for each selection cycle.

## Data Availability

The data presented in this study are openly available in FigShare at https://doi.org/10.6084/m9.figshare.12867062.v1, (accessed on 25 March 2021).

## Supplementary Materials

The following are available online at https://www.mdpi.com/article/10.3390/plants10091845/s1, Table S1: Phenotypic data of 98 genotypes evaluated in the environments of Campos dos Goytacazes (ENV1) and Itaocara (ENV2).Table S2: Genomic regions detected by regional heritability mapping (RHM) in 0.1 Mb genomic segments with a 0.05 Mb sliding window for traits grain yield (GY), kernel popping expansion (PE), plant height (PH), and ear height (EH) evaluated in a popcorn population under intrapopulation recurrent selection in the environments of Campos dos Goytacazes (ENV1) and Itaocara (ENV2).
