# Supplementary material for: Regional Heritability Mapping of Quantitative Trait Loci Controlling Traits Related to Growth and Productivity in Popcorn (Zea mays L.)
Source: Plants (Basel). 2021 Sep 6;10(9):1845. doi: 10.3390/plants10091845 (PMC8466968; doi:10.3390/plants10091845)
Supplement: Supplementary file 1 [file plants-10-01845-s001.zip › plants-1284221-supplementary.pdf]

Article

Regional heritability mapping of quantitative trait loci controlling traits related to growth and productivity in popcorn (*Zea mays* L.)

Gabrielle Sousa Mafra [1], Janeo Eustáquio de Almeida Filho [2], Antônio Teixeira do Amaral Junior [3,*], Carlos Maldonado [4], Samuel Henrique Kamphorst [3], Valter Jário de Lima [3], Divino Rosa dos Santos Junior [3], Jhean Torres Leite [3], Pedro Henrique Araujo Diniz Santos [3], Talles de Oliveira Santos [3], Rosimeire Barboza Bispo [3], Uéliton Alves de Oliveira [3], Vitor Batista Pinto [3], Alexandre Pio Viana [3], Caio Cezar Guedes Correa [3], Sunny Ahmar [5] and Freddy Mora-Poblete [5]

[1] Centro de Ciências Agrárias, Universidade Estadual da Região Tocantina do Maranhão, R. Godofredo Viana, 1300, Imperatriz, Maranhão, 65900-000, Brazil; gabrielle.smafra@yahoo.com.br (G.S.M.)

[2] Bayer, Estrada da Invernadinha, 2000, Coxilha 99145-000, Rio Grande do Sul, Brazil; janeo.filho@bayer.com (J.E.A.F.)

[3] Laboratory of Plant Breeding, Center of Agricultural Science and Technology, Darcy Ribeiro State University of Northern Rio de Janeiro, Av. Alberto Lamego, 2000, Campos dos Goytacazes, RJ 28013-602, Brazil; amaraljr@uenf.br (A.T.A.J); samuelkampho@hotmail.com (S.H.K.); valter_jario@hotmail.com (V.J.L.); juniorifagro@gmail.com (D.R.S.J.); torresjhean@gmail.com (J.T.L.); phsantos2004@yahoo.com.br (P.H.A.D.S.); tallesdeoliveira@live.com (T.O.S.); rosimeirebarboza1@hotmail.com (R.B.B.); uelitonalves2011@hotmail.com (U.A.O.); vvitorbp@gmail.com (V.B.P.); pirapora@uenf.br (A.P.V.); caiocagronomo@gmail.com (C.C.G.C.)

[4] Instituto de Ciencias Agroalimentarias, Animales y Ambientales, Universidad de O'Higgins, San Fernando 3070000, Chile; cmaldo1782@gmail.com (C.M.)

[5] Institute of Biological Sciences, University of Talca, 1 Poniente 1141, Talca, 3460000, Chile; sunnyahmar13@gmail.com (S.A); morapoblete@gmail.com (F.M-P.)

* Correspondence: amaraljr@uenf.br; Tel.: +55 22 999220192

Table S1. Phenotypic data of 98 genotypes evaluated in the environments of Campos dos Goytacazes, RJ (ENV1) and Itaocara (ENV2).

	ENV1				ENV2			
**Genotype**	**EH (cm)**	**GY (Kg/ha)**	**PE (mL/g)**	**PH (cm)**	**EH (cm)**	**GY (Kg/ha)**	**PE (mL/g)**	**PH (cm)**
1	73.27	1960.87	33.11	149.56	102.39	2288.52	27.20	190.77
2	99.41	1947.64	27.29	186.43	126.18	1281.50	28.16	205.82
3	69.29	2814.74	26.51	157.56	100.62	2658.59	26.25	193.33
4	97.38	2594.04	25.57	178.32	123.69	1886.50	26.65	207.16
5	72.99	1691.61	29.39	160.08	86.35	1972.00	27.55	196.42
6	75.44	2500.66	28.98	159.10	104.71	1603.03	24.62	197.14
7	79.83	1891.93	27.79	153.76	99.72	2201.44	29.15	194.23
8	92.83	1688.79	25.11	188.93	125.11	1479.31	25.38	231.80
9	76.76	1649.44	27.78	176.59	93.92	1748.82	26.49	193.57
10	84.70	1526.39	29.07	172.76	111.67	1232.90	23.15	198.58
11	92.07	2904.26	30.10	171.66	128.93	1023.99	24.72	223.41
12	90.57	2720.42	31.57	191.54	113.79	2262.23	28.47	223.33
13	68.66	2219.72	28.57	154.34	90.09	2310.89	28.94	181.62
14	98.23	3793.40	27.02	185.66	130.28	3152.86	28.70	232.96
15	88.01	1909.87	27.28	180.25	117.92	2030.17	31.37	214.01
16	90.33	2856.07	30.12	173.47	122.87	2551.50	29.01	221.91
17	80.62	3760.85	28.81	171.53	116.31	2216.27	26.41	207.35
18	80.67	2894.96	33.25	146.35	103.18	1262.58	33.52	186.27
19	65.23	2715.90	28.80	139.12	85.08	1541.05	28.89	158.79
20	81.56	2819.93	36.07	161.46	112.07	1379.23	30.88	198.67
21	101.07	2695.13	24.35	180.04	161.12	3238.11	26.08	244.93
22	76.90	3109.69	33.21	157.40	106.06	2107.30	28.50	193.07
23	95.07	2079.03	28.43	166.17	129.52	1601.21	30.38	199.09
24	73.91	2251.91	26.17	150.51	101.91	1651.46	24.12	181.87
25	86.59	3335.70	29.78	176.47	118.32	2565.16	26.78	207.91
26	83.22	2313.83	26.47	163.05	117.50	1878.21	29.64	210.11
27	79.59	2461.80	29.49	159.25	110.03	1861.39	23.72	205.85
28	98.52	1978.74	30.29	181.94	144.28	2246.22	23.92	236.18
29	88.56	2685.53	28.47	173.46	113.45	1828.60	30.48	208.83
30	91.66	1983.79	30.53	175.89	125.97	1876.48	34.63	208.59
31	80.55	2093.45	31.02	171.07	107.65	1708.77	26.98	213.20
32	81.36	2234.59	27.67	174.14	97.95	1303.36	29.50	190.22
33	90.79	2868.30	23.97	176.63	138.16	1789.38	20.33	229.50
34	88.14	2710.55	25.30	170.58	124.30	2719.08	31.01	220.17
35	77.27	2432.86	27.04	153.37	112.86	2410.21	20.27	190.81
36	77.97	2715.28	32.89	156.23	116.46	2260.05	30.11	201.51
37	70.40	2620.29	28.85	143.59	105.00	2122.68	30.51	180.59
38	76.90	1986.09	25.29	160.16	107.30	1886.87	24.46	199.30
39	85.40	2557.40	34.07	164.79	114.25	2516.20	29.04	200.68
40	81.47	1955.81	31.13	163.23	117.50	1347.83	28.54	210.10
41	73.95	2597.70	27.30	158.36	116.64	2218.88	30.82	201.73
42	83.30	2846.83	21.43	165.45	117.74	2705.26	25.16	203.25

	ENV1				ENV2			
Genotype	EH (cm)	GY (Kg/ha)	PE (mL/g)	PH (cm)	EH (cm)	GY (Kg/ha)	PE (mL/g)	PH (cm)
43	79.01	2203.62	27.36	143.40	103.92	1159.42	27.44	187.33
44	90.93	2816.52	24.34	170.99	135.46	2797.92	24.59	226.16
45	74.61	3272.61	29.00	161.09	93.82	1981.07	22.49	183.48
46	81.23	2515.24	28.34	169.21	102.07	2039.56	31.21	202.23
47	70.94	1894.92	31.91	163.91	105.47	1861.14	32.60	214.90
48	89.35	2604.99	26.29	168.51	132.92	1763.73	28.77	214.02
49	77.11	2239.41	26.40	154.74	96.08	1782.57	27.93	180.23
50	90.80	1984.17	29.68	179.25	129.90	1969.12	28.33	210.24
51	96.07	2310.52	26.14	167.76	119.14	2972.61	27.25	209.90
52	97.07	2142.61	27.17	181.71	122.20	1568.59	17.42	210.74
53	72.93	2552.32	32.45	146.85	100.57	2015.99	30.94	178.51
54	83.74	2310.93	25.77	164.35	108.74	2096.08	25.23	189.42
55	87.93	2434.05	32.79	166.12	117.05	1983.19	29.13	209.10
56	80.32	2381.57	30.64	169.63	106.34	2059.80	26.14	204.53
57	81.33	1865.46	21.15	178.29	109.05	2153.05	22.34	213.51
58	70.73	2634.23	30.89	136.32	93.90	1458.80	34.20	177.61
59	80.75	2707.12	24.89	168.18	104.31	1201.81	20.56	197.51
60	85.46	3332.27	24.16	170.36	121.78	2143.82	28.09	213.32
61	82.57	2306.09	22.73	165.20	122.20	2126.99	26.64	214.25
62	89.68	2692.52	35.15	174.84	125.74	2375.44	33.05	210.92
63	79.58	2064.87	27.15	158.27	95.91	2813.77	28.75	189.10
64	85.64	2694.07	26.33	173.38	115.30	1901.08	26.42	201.55
65	73.93	2635.57	29.24	146.07	104.53	2621.21	26.94	188.31
66	106.18	4681.56	27.24	198.41	142.57	1999.99	29.20	235.62
67	88.96	2894.12	26.04	177.87	122.85	3357.39	27.39	220.51
68	91.45	2309.89	27.98	167.74	115.60	1482.42	28.14	208.51
69	82.04	3388.20	28.24	159.10	107.09	1564.48	25.61	197.81
70	81.33	2908.68	26.50	155.72	100.54	2870.83	26.71	189.07
71	90.38	2282.51	30.46	161.68	105.94	1395.49	30.18	192.76
72	86.07	2225.82	30.07	161.98	114.41	2438.42	32.62	203.41
73	89.66	2642.13	28.31	169.57	106.33	2395.88	24.07	192.53
74	90.14	2576.69	34.88	182.98	117.38	1788.49	28.82	213.22
75	73.29	3213.82	32.55	162.34	108.95	1735.64	26.86	212.48
76	98.35	2671.02	29.89	191.75	126.46	3017.48	31.31	225.93
77	89.90	2252.92	29.01	174.01	122.22	2277.87	26.58	221.26
78	89.32	2417.23	29.72	184.24	114.01	984.91	25.46	225.55
79	84.55	2179.86	24.29	172.41	110.20	2387.51	26.96	207.58
80	87.91	3136.64	32.86	174.89	122.59	2772.15	30.82	219.66
81	68.72	2500.56	25.78	144.78	98.32	2403.19	23.40	171.53
82	82.92	1794.03	24.38	158.43	113.16	1507.33	26.52	193.72
83	77.96	2608.80	31.16	159.56	88.95	2408.49	33.98	183.92
84	89.59	2130.82	32.06	176.26	119.59	2559.25	26.22	206.19
85	83.39	2933.70	28.12	166.73	101.02	3109.62	25.48	188.91
86	84.83	2624.59	30.88	162.64	121.83	3263.77	30.87	206.36

	ENV1				ENV2			
**Genotype**	**EH (cm)**	**GY (Kg/ha)**	**PE (mL/g)**	**PH (cm)**	**EH (cm)**	**GY (Kg/ha)**	**PE (mL/g)**	**PH (cm)**
87	87.96	2550.33	29.47	165.54	123.83	2876.12	29.33	212.66
88	79.45	2329.22	32.08	169.49	106.31	1761.34	30.04	212.05
89	73.79	1346.22	29.37	145.53	110.82	1049.97	28.82	180.51
90	99.86	3160.99	31.81	187.78	144.36	2691.64	31.86	227.23
91	79.63	3091.60	29.84	156.13	101.00	2359.75	25.79	196.69
92	82.98	2264.27	28.11	159.44	108.37	2120.05	26.52	199.85
93	85.46	3572.66	32.46	172.64	120.33	3086.75	29.44	198.22
94	87.18	1971.89	27.31	172.08	118.13	1858.36	28.68	207.34
95	94.96	2449.99	27.95	181.88	133.55	2526.29	28.36	226.55
96	76.99	2401.83	28.85	153.98	105.74	2934.51	30.87	192.69
97	96.31	3579.23	30.52	186.78	130.83	2235.69	31.55	241.43
98	83.11	1938.13	26.25	169.98	104.21	1800.46	24.33	171.66

Table S2. Genomic regions detected by regional heritability mapping (RHM) in 0.1 Mb genomic segments with a 0.05 Mb sliding window for traits grain yield (GY), kernel popping expansion (PE), plant height (PH) and ear height (EH) evaluated in a popcorn population under intrapopulation recurrent selection in the environments of Campos dos Goytacazes, RJ (ENV1) and Itaocara (ENV2).

ENV	Trait	Chrom.	Inital position of the region (base pairs)	Final position of the region (base pairs)	Number of SNPs in the region	h^2REG	$-log_{10}$ p
ENV1	EH	2	148888849	148988849	4	0.08	3.17
ENV1	EH	2	148838849	148938849	7	0.08	3.03
ENV1	GY	1	48905387	49005387	2	0.13	3.85
ENV1	GY	1	48955387	49055387	2	0.13	3.85
ENV1	GY	1	56805387	56905387	2	0.49	3.67
ENV1	GY	1	56855387	56955387	2	0.49	3.67
ENV1	GY	6	165623194	165723194	5	0.93	3.47
ENV1	GY	2	225088849	225188849	7	0.11	3.32
ENV1	GY	2	225138849	225238849	7	0.11	3.32
ENV1	GY	4	219528603	219628603	2	0.29	3.27
ENV1	GY	4	219578603	219678603	2	0.29	3.27
ENV1	PE	2	13388849	13488849	8	0.56	4.20
ENV1	PE	2	13438849	13538849	8	0.56	4.20
ENV1	PE	5	13560296	13660296	7	0.20	3.17
ENV1	PE	5	13610296	13710296	7	0.20	3.17
ENV1	PH	8	171723438	171823438	3	0.11	3.08
ENV1	PH	8	171773438	171873438	3	0.11	3.08
ENV2	EH	7	115031917	115131917	2	0.12	3.12
ENV2	EH	7	115081917	115181917	2	0.12	3.12
ENV2	EH	4	178478603	178578603	9	0.14	3.12
ENV2	GY	2	200838849	200938849	3	0.08	3.28
ENV2	GY	2	200888849	200988849	3	0.08	3.28
